# Positive Influence of Liquid Sodium Silicate on the Setting Time, Polymerization, and Strength Development Mechanism of MSWI Bottom Ash Alkali-Activated Mortars

**DOI:** 10.3390/ma14081927

**Published:** 2021-04-12

**Authors:** Lei Jin, Guodong Huang, Yongyu Li, Xingyu Zhang, Yongsheng Ji, Zhishan Xu

**Affiliations:** 1 School of Civil Engineering and Construction, Anhui University of Science and Technology, No 168, Taifeng Road, Huainan 232001, China; leijin@aust.edu.cn (L.J.); yyli@aust.edu.cn (Y.L.); xyzhang@aust.edu.cn (X.Z.); 2 School of Energy and Safety, Anhui University of Science and Technology, No 168, Taifeng Road, Huainan 232001, China; 3 School of Mechanics and Civil Engineering, China University of Mining and Technology, Xuzhou 21116, No 1, Daxue Road, Xuzhou 221116, China; jiyongsheng@cumt.edu.cn (Y.J.); xuzhishan@cumt.edu.cn (Z.X.)

**Keywords:** liquid sodium silicate, setting time, municipal solid waste incineration bottom ash, compressive strength, alkali-activated mortar

## Abstract

Setting time and mechanical properties are key metrics needed to assess the properties of municipal solid waste incineration (MSWI) bottom ash alkali-activated samples. This study investigated the solidification law, polymerization, and strength development mechanism in response to NaOH and liquid sodium silicate addition. Scanning electron microscopy and X-ray diffraction were used to identify the formation rules of polymerization products and the mechanism of the underlying polymerization reaction under different excitation conditions. The results identify a strongly alkaline environment as the key factor for the dissolution of active substances as well as for the formation of polymerization products. The self-condensation reaction of liquid sodium silicate in the supersaturated state (caused by the loss of free water) is the major reason for the rapid coagulation of alkali-activated samples. The combination of both NaOH and liquid sodium silicate achieves the optimal effect, because they play a compatible coupling role.

## 1. Introduction

The reduction, harm-reduction treatment, and recycling of municipal solid waste (MSW) is a hot issue both in China and internationally [1]. To alleviate problems associated with the disposal of harmful MSW, power generation via incineration has become an important means to dispose of MSW. This not only uses the heat energy of burning MSW to generate electricity, but also realizes the required reduction of the MSW volume. The products of MSW incineration can mainly be divided into two categories: (1) fly ash and (2) bottom ash (BA) [2,3]. BA accounts for ~80% of the total incineration products and is mainly composed of different types of large particles (45 mm), including molten slag blocks, glass, rocks, and small metal fragments [4]. The yield of BA is enormous, and relevant data indicate that China’s annual output of BA has reached 5 billion tons in 2019 [5]. Although the incineration of MSW can better achieve volume reduction treatment, it cannot achieve the complete purpose of environmental harmless treatment, let alone the recycling and utilization of MSW [6]. Not only the yield of BA is enormous, but also, the recycling technology is challenging. Therefore, only by completely realizing both the recycling and utilization of BA, can the problems caused by MSW accumulation be completely eliminated.

BA is mainly composed of elements with high-boiling points, in which the accumulated contents of silica, alumina, iron oxide, and calcium oxide account for about 80%, while inorganic compounds with low volatility are also concentrated [7]. More importantly, BA is rich in active polymerization reaction components (i.e., Ca^2+^, Si^4+^, and A1^3+^), high bond strength of which can be established under polymerization of active components in strong alkaline environment [8,9]. An alkali-activated material is a kind of inorganic non-metallic material, which mostly uses industrial solid waste as raw materials that is rich in silicate and silicoaluminate minerals. Under the strongly alkaline environment, active substances in the raw materials are quickly dissolved and polymerized, thus generating calcium silicate hydrate and calcium aluminate silicate hydrate and gradually developing high strength. In terms of material properties, alkali-activated materials have characteristics of high strength, high temperature resistance, acid, alkali, and salt corrosion resistance, as well as excellent resistance to permeability and wear compared with Portland cement [10]. During production and preparation processes, alkali-activated materials use industrial solid waste as the main raw material, do not require high temperature calcination, and after treatment, the raw materials can be recycled again [11]. Therefore, using alkali-activated BA materials provides a feasible way for the large-scale and safe absorption and recycling of BA.

Several previous studies investigated the polymerization and strength development mechanisms underlying alkali-activated slag and related setting and hardening mechanisms [12,13]. Provis et al. [14,15] systematically studied the mechanical properties and relative durability of alkali-activated materials. Bernal et al. [16,17] reported that the mechanical properties of concrete made of alkali-activated slag are better than those of concrete based on Portland cement. With increasing binder content, the permeability, water absorption, and carbonation resistance of alkali-activated slag concrete can be further improved. These results are coherent with those reported by Shi et al. [18,19], who suggested that the amount of alkali and sodium silicate in initiators greatly influences the mechanical properties and durability of alkali-activated slag mortars. However, Zhang et al. [20,21] reported that adding too much alkali and sodium silicate to alkali-activated slag mortars will cause a strong alkali silicon reaction, which has adverse effects on the development of both mechanical properties and durability. Therefore, the effects of sodium silicate and strong alkali on the strength development and polymerization mechanism of alkali-activated samples are likely distinct [22]. Both the sodium silicate and strong alkali seem to exert coupling effects on the condensation characteristics and polymerization reaction of alkali-activated samples [23]. This is certainly a question worthy of further exploration; however, the effect of both strong alkali and sodium silicate on the setting time and condensation mechanism of BA have not been investigated to date. Both strong alkali and sodium silicate exert synergistic coupling effects on the dissolution of active substances dissolved from raw materials and the formation of polymerization products with high strength.

This work investigated the effect of both liquid sodium silicate and NaOH on the setting and hardening mechanism, polymerization mechanism, and strength development mechanism of BA alkali-activated mortars. The influence on the time-varying law of condensation and hardening as well as the strength-development mechanism has been deeply studied in response to the addition of different dosages of NaOH, solid sodium silicate, and liquid sodium silicate. A polymerization mechanism model of the dissolution of active substances and the formation of polymerization products was established. This model mainly considered the influence of NaOH and sodium silicate contents. Scanning electron microscopy (SEM) and X-ray diffraction (XRD) were used to further verify the condensation and polymerization reaction mechanisms.

## 2. Experimental Materials and Methods

### 2.1. Materials

#### 2.1.1. Bottom Ash

BA was provided by the Wanneng environmental protection electric power Co., Ltd., Huainan, Anhui, China. The MSW has been calcined at temperatures exceeding 900 °C. BA has been processed by magnetic separation followed by water washing in the recycling station; the recyclable metals were recycled and soluble hazardous substances were disposed of. To improve the reaction activity, BA should be first dried and then ball ground prior to the experiment, for which the specific surface area is 416 m^2^/kg. The BA particles have a median particle size of 49 μm and 32% was retained on a 45 μm sieve. The chemical components of BA, which were obtained by X-ray fluorescence spectrum analysis (XRF), are shown in Table 1.

#### 2.1.2. Ground Granulated Blast Furnace Slag

Ground granulated blast furnace slag (GGBFS) was provided by Huihao environmental protection technology Co., Ltd., Hebei, China. The main purpose of the incorporation of GGBFS was to further enhance the reactivity of BA. Its performance meets the national standard of China (S95 Ground granulating blast furnace slag used for cement and concrete, GB18046-2008) [24]. The specific surface area of GGBFS is 430 m^2^/kg. The BA particles have a median particle size of 47 μm and 24% was retained on a 45 μm sieve. Table 1 also shows the chemical composition of GGBFS, which was identified via XRF.

#### 2.1.3. Initiator

This experiment used both liquid sodium silicate (Na_2_SiO_3_) and sodium hydroxide (NaOH) as initiator. The order of their addition and the exact method is presented in Section 2.2.2 and the specific dosages are shown in Table 2.

Sodium hydroxide (NaOH, flake, purity 95%) was provided by Cangzhou Rongqing Chemical Co., Ltd., Hebei, China. Liquid sodium silicate consisted of 9.67% Na_2_O, 25.14% SiO_2_, and 65.11% H_2_O, and was provided by Wuxi Yanxiang Chemical Materials Co., Ltd., Jiangsu, China.

#### 2.1.4. Others

ISO standard sand (fineness modulus 2.76) was used as fine aggregate [25] and the test used tap water. The specific mixing amount of sand and test water is shown in Table 2.

### 2.2. Methods

#### 2.2.1. Mix Proportions of Bottom Ash Alkali-Activated Mortars

Table 2 presents the mix proportion of samples. All mortar samples adopted the same liquid–solid ratio and mix proportion but differed in the kinds and amounts of initiators. To the control group A-1, neither liquid sodium silicate, nor sodium hydroxide was added. Groups from A-2 to A-3 were excited by NaOH at different dosages to explore the influence of the NaOH content on the setting time and strength development of BA alkali-activated materials. Groups from A-4 to A-6 were excited by different dosages of liquid sodium silicate, to explore its effect on coagulation acceleration and strength development. Finally, groups from A-7 to A-9 were excited by both liquid sodium silicate and NaOH to explore the coupling and promoting effect of mixed initiators.

#### 2.2.2. Preparation and Curing Process of Samples

When BA comes in contact with sodium hydroxide, serious foaming, and expansion will occur, which seriously affected the development of the mechanical properties of samples. Therefore, it is necessary to mix BA with sodium hydroxide and test water (Table 2) to form a slurry as an anti-foam pretreatment. This was followed by 3 h of defoaming pre-treatment before mortar preparation. Then, the treated slurry and GGBFS were added to a blender and first mixed slowly for 30 s and then quickly for 30 s. Finally, sand and liquid sodium silicate were added and quickly stirred for 1 min and the mixture was then poured into standard sand sample molds (40 mm × 40 mm × 160 mm) [25].

After casting, samples were immediately put into the curing room, and the mold was removed after curing for 24 h. During curing, the temperature of the curing room was maintained at 20 ± 2 °C and the humidity exceeded 95% [25].

#### 2.2.3. Compressive Strength Tests

When the samples were cured to a specified age (3 days, 28 days, and 60 days), MTSY-300 type mortars compressive strength constant stress press (provided by Hebei Sansi Test Equipment Technology Co., Ltd., Zhejiang, China) was used to measure the compressive strength of samples. The test method was in accordance with the method of testing cements—determination of strength (ISO, GB/T17671-1999) [25]. Test results are presented as the arithmetic mean values of the six compressive strength values obtained from three samples.

#### 2.2.4. Initial and Final Setting Time Tests

The initial and final setting times of paste samples were measured by a standard Vicat apparatus (provided by Hebei Shengke Test Equipment Technology Co., Ltd., Cangzhou, China) in reference to the apparatus for determining normal consistency and setting time of cement paste (JC/T 727-2005) [26]. During the test, the truncated taper with an upper circle diameter of 65 mm, a lower circle diameter of 75 mm, and a depth of 40 mm was adopted.

The specific testing process consisted of three steps: (1) Paste samples were prepared in reference to the casting method described in Section 2.2.2 and the mixing ratio (see Table 2) to prepare paste samples. The water–binder ratio of paste samples was 0.3, which represents water of normal consistency, as determined experimentally. The initial setting time was defined as the time-point when GGBFS was added to the slurry. (2) Determination of initial setting time: when the test needle sinks to 3–5 mm from the bottom plate, which means that the paste sample reaches the initial setting state, denoted by ‘min’. The initial setting time of paste samples was defined as the time when all GGBFS was added to the slurry at the initial setting state. The initial setting time results were reported as the averages of three samples. (3) Determination of the final setting time: the test mold and slurry were turned 180°, and the big end was placed up and the small end down on a glass plate. The time when the test needle sinks into the test body for 0.5 mm—i.e., when the annular attachment (which tests the pointer of the final setting time) failed to leave traces on the test body at the beginning—was defined as the final setting state of the sample. The final setting time of the paste sample was defined as the time when all GGBFS was added to the slurry to achieve the final setting state. The reported results of the final setting time are the average of three samples.

#### 2.2.5. Microstructural Analysis

(1) SEM Analysis

To study the microstructure, component characteristics, and the condensation mechanism of alkali-activated paste samples under microscopic conditions, the paste samples A-3, A-5, A-8, and A-9 were prepared for SEM analysis. A high-resolution field emission scanning electron microscope (MAIA3 LMH, Tescan, Brno, Czech Republic) was used. The amplification factor was 4–1,000,000 times, the acceleration voltage was 200 V to 30 kV in decelerating mode, and the probe current was 2 PA-400 nA.

(2) X-ray Diffraction Analysis

To study the crystal structure, phase composition, and its variation rule in alkali-activated paste samples under different excitation conditions, paste samples A-3, A-5, and A-8 were prepared for XRD analysis. The powder crystal X-ray diffractometer (D8 Advance, Bruker, Bremen, Germany) consists of a closed ceramic tube X-ray source, an X-ray high pressure generator, a high-precision wide-angle goniometer, a highly sensitive links array detector, a cooling water system, and a computer to control the instrument and process the data. Measurement range (2θ) was 5–70°.

## 3. Results and Discussion

### 3.1. Mechanical Properties of Bottom Ash Alkali-Activated Mortars

#### 3.1.1. Effect of NaOH on Compressive Strength

**Figure 1:**
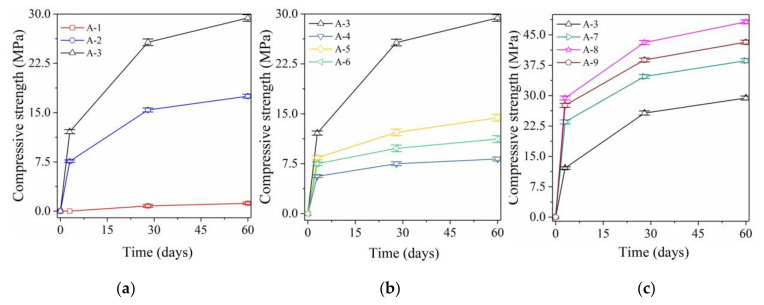
Compressive strength of samples excited by different initiator. (**a**) Excited by NaOH; (**b**) excited by sodium silicate; (**c**) excited by both NaOH and sodium silicate.

Figure 1 shows how the development trend of compressive strength of alkali-activated mortars with the same mix ratio but different initiators.

Figure 1a shows the compressive strength of mortars with different amounts of NaOH as initiator. The compressive strength of sample A-1 was clearly insufficient and the molds could not be removed after curing for 3 days. The strengths of sample A-1 were still only 0.8 MPa (28 days) and 1.2 MPa (60 days), respectively, which does not represent a significant increase. This suggests that BA and GGBFS have very low binding ability and cannot effectively establish strength without strong alkali excitation (Table 2, pH = 10.12). With the addition of NaOH (12 g), the pH of the initiator increased from 10.12 (A-1) to 14.12 (A-2) and the compressive strength of sample A-2 increased to 7.6 MPa (3 days), 15.4 MPa (28 days), and 17.5 MPa (60 days). With further addition of NaOH (24 g), the pH of the initiator in sample A-3 further increased to 14.43 and the compressive strength further increased to 12.1 MPa (3 days), 25.7 MPa (28 days), and 29.4 MPa (60 days). This represents a significant increase compared with sample A-2. This indicates that the highly alkaline excitation environment is more conducive to the polymerization of active calcium, silicon, and aluminum as well as the strength development.

#### 3.1.2. Effect of Liquid Sodium Silicate on Compressive Strength

Figure 1b shows the compressive strengths of mortars, which used liquid sodium silicate (100 g, 120 g, and 140 g) as initiator. When the type of initiator changed from NaOH to liquid sodium silicate (100 g), the pH of the initiator in sample A-4 decreased to 11.55 and the compressive strength decreased to 5.6 MPa (3 days), 7.5 MPa (28 days), and 8.2 MPa (60 days). This represents a significant decrease compared with sample A-3. Addition of liquid sodium silicate achieved rapid condensation of samples and established a certain strength in a low-alkalinity environment, which will be discussed in detail in Section 3.2. However, a lack of high pH in the excitation environment still cannot promote the required strength development nor lead to the high strength properties of alkali-activated materials. When the liquid sodium silicate content increases from 100 g to 120 g, the pH of the initiator in sample A-5 did not increase, but the compressive strength increased to 8.4 MPa (3 days), 12.2 MPa (28 days), and 14.4 MPa (60 days). This represents a slight increase compared with sample A-4. However, further increasing the liquid sodium silicate content (from 120 g to 140 g) even caused a slight decrease in the compressive strength of sample A-6. Therefore, the excitation effect of liquid sodium silicate was significantly lower than that of sodium hydroxide. With increasing liquid sodium silicate content, the compressive strength of samples first increased slightly and then decreased.

#### 3.1.3. Effect of Both NaOH and Sodium Silicate on Compressive Strength

Figure 1c shows the compressive strengths of alkali-activated mortars, which used different amounts of both liquid sodium silicate and NaOH as initiators. The compressive strengths of sample A-7 (Table 2, pH = 14.49) increased to 34.7 MPa (28 days) and 38.6 MPa (60 days), representing increases of 35% and 31.3%, respectively, compared with sample A-3. If liquid sodium silicate and NaOH are used in combination as initiators, the stimulation effect is good, the condensation of the sample is promoted, and the strength development is accelerated. Keeping the sodium hydroxide content constant (24 g) and increasing the liquid sodium silicate content (100 g to 120 g), the compressive strength of sample A-8 reached up to 43.1 MPa (28 days) and 48.2 MPa (60 days), representing increases by 67.7% and 63.9%, respectively. This represents a further significant increase compared with sample A-3. The increased liquid sodium silicate content provides more active sodium and silicon. Moreover, sodium hydroxide (24 g) provides an excitation environment with sufficiently high pH, thus further promoting the improvement of compressive strength [27]. However, the compressive strength of sample A-9 did not further improve (as expected), but rather decreased with further increases of liquid sodium silicate content (from 120 g to 140 g). Simply increasing the content of reactive silicon in the polymerization reaction environment will not lead to a continuous increase of the compressive strength.

### 3.2. Effects of Different Initiators on the Setting Time

#### 3.2.1. Effect of NaOH on the Setting Time

**Figure 2:**
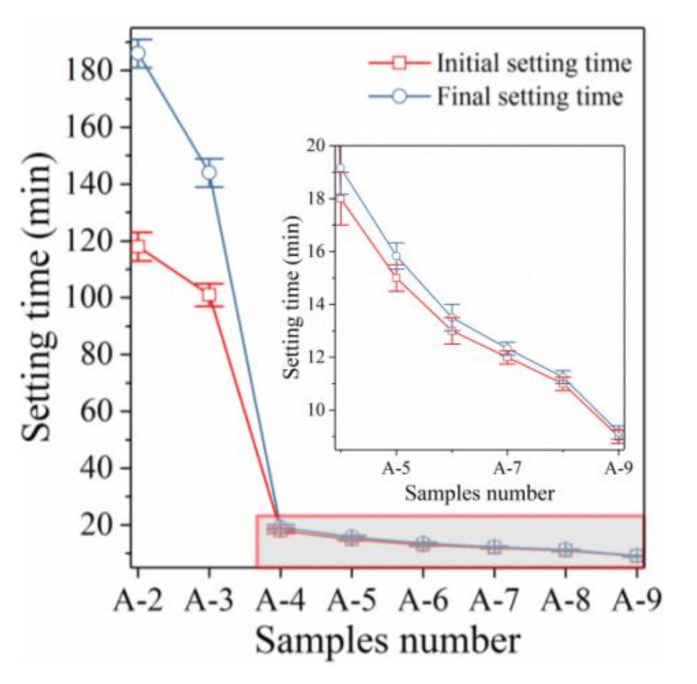
Effect of different initiator on initial/final setting time.

The influences of different initiators on the initial and final setting times of samples and the relevant rules are shown in Figure 2 and Table 3.

When neither NaOH nor liquid sodium silicate was added, the pH of the initiator only reached 10.02 (Table 2) and sample A-1 did not reach the initial setting state even after 10 h. This suggests that in the absence of a strong alkaline excitation environment (established by NaOH), the mixed binding materials of BA and GGBFS have no binding ability and did not manage to coagulate the alkali-activated sample A-1. With increasing NaOH content, the alkali-activated samples began to coagulate and the initial setting time was continuously shortened (see Figure 2 red line) and the compressive strength also began to increase (Figure 1). When 12 g NaOH was added, the pH of the initiator in sample A-2 achieved a remarkable increase (Table 2, from 10.02 to 14.12), the initial setting time of sample A-2 was 118 min, and the final setting time was 186 min. This suggests that the binding material (BA and GGBFS) can only be properly activated under the condition of a pH above 14. This highly alkaline excitation environment (pH 14) achieved the condensation of samples and promoted the strength development. However, the final setting time of sample A-2 was clearly shorter (only 118 min) and the time interval between the initial setting and final setting was only 68 min, which was significantly lower than that of Portland cement (which is 300 min). With further increasing NaOH content, the initial setting time of the alkali-activated sample was continuously shortened and the time interval between the initial setting and the final setting was also continuously shortened, accompanied by an obvious increase of the compressive strength. When the NaOH content was increased from 12 g to 24 g, the pH of the initiator in sample A-3 also showed a further significant increase (Table 2, from 14.12 to 14.43). The initial setting time of sample A-3 decreased to 101 min, and the final setting time was shortened to 144 min. This suggests that further increasing the pH of the polymerization reaction environment can indeed further enhance the activity of BA and GGBFS and thus accelerate the coagulation of alkali-activated samples and promote the development of compressive strength [28].

#### 3.2.2. Effect of Sodium Silicate on the Setting Time

When the initiator is changed from sodium hydroxide to liquid sodium silicate, the coagulation characteristics of alkali-activated samples changed completely. When the liquid sodium silicate content reached 120 g, the pH of the polymerization environment in sample A-4 decreased to 11.55 (Table 2). Rapid condensation was achieved in the low-alkalinity environment, the initial setting time decreased to 1080 s, and the final setting time was only 1150 sec. The setting time was significantly lower than that of NaOH and the initial coagulation and final coagulation occurred almost simultaneously. Liquid sodium silicate can achieve rapid condensation of alkali-activated samples under low alkalinity (pH = 11.55) but it cannot effectively promote the growth of compressive strength (Figure 1). However, it is necessary to establish an environment with high pH (pH 14) when NaOH is used as initiator to realize the condensation of alkali-activated samples; however, such a highly alkaline environment can effectively promote the strength development. This shows that the condensation mechanism of both NaOH and liquid sodium silicate on alkali-activated samples is completely different. NaOH stimulates the dissolution and promotes the polymerization of the active substances in BA and GGBFS by forming a strong alkali excitation environment. This provides favorable conditions for the fusion of active substances and the strength development of samples [29]. However, liquid sodium silicate provides a much less alkaline excitation environment (pH = 11.55) than sodium hydroxide (pH 14). This cannot form an effective excitation environment to induce the dissolution of reactive calcium, silicon, and aluminum from BA and GGBFS and the formation of polymerization products. Only the self-condensation ability of liquid sodium silicate leads to the coagulation of alkali-activated samples [30]. This explains why rapid coagulation occurred in sample A-4, while the compressive strength did not increase significantly. The condensation mechanism of alkali-activated samples will be presented in detail in Section 3.4. With increasing liquid sodium silicate content, the initial and final setting times of samples were further shortened. Moreover, the time interval between initial and final setting continuously shortened, while the compressive strength did not increase at all. When the liquid sodium silicate content reached 140 g, the initial and final setting times of sample A-6 were 780 and 810 sec, respectively. Increasing the liquid sodium silicate content further accelerated the coagulation of alkali-activated samples and initial setting and final setting almost happened at the same time.

#### 3.2.3. Effect of Both NaOH and Sodium Silicate on the Setting Time

When a mixture of NaOH and liquid sodium silicate was used as initiator, the setting time of alkali-activated samples was further shortened, and the compressive strength increased significantly. When the liquid sodium silicate content reached 100 g and the amount of NaOH was 24 g, the initial and final setting times of sample A-7 were 720 and 740 sec, respectively. With further increasing liquid sodium silicate content, the initial and final setting times of sample A-9 further decreased to 540 and 550 sec, respectively. The excitation effect of the NaOH and liquid sodium silicate mixture is better than that of either initiator individually. Liquid sodium silicate first achieved rapid condensation and the alkali-activated sample first polymerized into a gel as a whole. Then, the high alkalinity environment formed by NaOH excites the active substances in BA and GGBFS to dissolve and polymerize on the gel structure, thus achieving the continuous densification of the structure of the gel and a continuous increase of the strength [31]. Moreover, the high alkalinity environment created by NaOH did not affect the rapid condensation characteristics of liquid sodium silicate, and also did not further promote the condensation of the alkali-activated sample. The rapid coagulation characteristics of liquid sodium silicate do not affect the excitation effect of NaOH, but enhance the polymerization reactivity of BA and GGBFS, and thus further promote the development of strength [32]. Therefore, both NaOH and liquid sodium silicate have a complementary excitation effect.

### 3.3. Results of Microanalysis

#### 3.3.1. SEM Analysis

**Figure 3:**
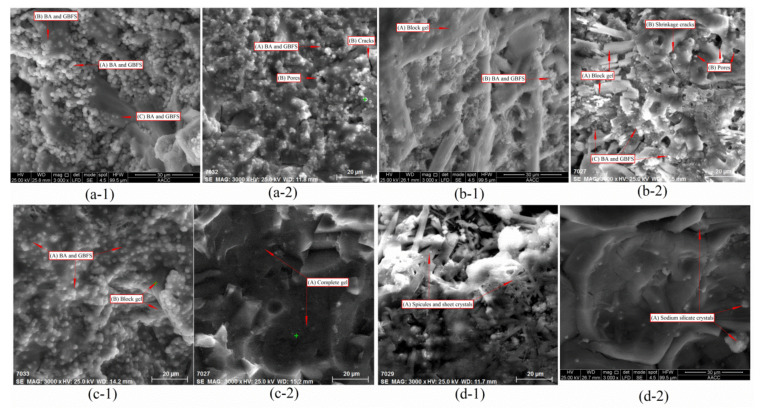
SEM (Scanning electron microscopy) analysis of paste samples A-3, A-5, A-8, and A-9; (**a-1**,**b-1**,**c-1**,**d-1**) 3 h after preparation of samples A-3 A-5, A-8, and A-9; (**a-2**,**b-2**,**c-2**,**d-2**) 28 d after preparation of samples A-3 A-5, A-8, and A-9.

The influences of different initiators on the degree of polymerization and microstructural changes of paste samples A-3, A-5, A-8, and A-9 are shown in Figure 3.

Figure 3(a-1) shows the microscopic reaction characteristics of sample A-3 after 3 h of NaOH excitation. A large number of round particles (BA and GGBFS) still remain loosely scattered in sample A-3 (as shown in (A) of Figure 3(a-1)), implying that the participation rate of particle polymerization reaction between BA and GGBFS is low. Moreover, several particles (BA and GGBFS) assembled and were embedded in the gels, suggesting that these particles have begun to participate in polymerization and have become part of the gels (as shown in (B) of Figure 3(a-1)). Only few particles (BA and GGBFS) have fully participated in the polymerization reaction and formed a complete gel (as shown in (C) of Figure 3(a-1)). Therefore, under NaOH excitation, the polymerization reaction rate of sample A-3 was very slow, and a large number of BA and GGBFS particles still did not participate in the reaction, even after 3 h. Figure 3(a-2) shows the microscopic reaction characteristics of sample A-3 after 28 d, excited by NaOH only. After 28 days of polymerization, BA and GGBFS particles scattered on the surface of the sample A-3 without polymerization had completely disappeared. However, a large number of particles still failed to participate in the polymerization, and were embedded on the surface of sample A-3, where they remained in a partial reaction state (as shown in (A) of Figure 3(a-2)). Moreover, many defects (pores and cracks) were also found in sample A-3 (as shown in (B) of Figure 3(a-2)), indicating that the degree of polymerization was low under NaOH excitation.

Figure 3(b-1) shows the microscopic reaction characteristics of sample A-5 after 3 h, which was only excited by liquid sodium silicate. In contrast to NaOH excitation, the surface of sample A-5 was completely covered by block gel after 3 h of polymerization. The uneven gels bonded to each other and completely covered sample A-5 (as shown in (A) of Figure 3(b-1)). However, a large number of particles (BA and GGBFS, as shown in (B) of Figure 3(b-1)) were not involved in the polymerization and can still be observed below the block gel. It is impossible for BA and GGBFS to form large amounts of gels in an environment with low pH and during short polymerization (3 h) [13]. This suggests that bulk gels are not produced by the polymerization of BA and GGBFS but are mainly formed by the self-condensation of liquid sodium silicate. In response to the loss of free water, liquid sodium silicate condensed and formed a large number of gels, covered with BA and GGBFS particles within a short time (less than 20 min) [33]. This is why the sample A-5 coagulated so rapidly; the condensation mechanism of liquid sodium silicate will be discussed in detail in Section 3.4. Figure 3(b-2) shows the microscopic reaction characteristics of sample A-5, which was only excited by liquid sodium silicate after 28 d. The surface of sample A-5 is still covered by a large volume of gel, which formed by self-condensation of liquid sodium silicate (as shown in (A) of Figure 3(b-2)). However, after 28 days of curing, loss of water caused by drying leads to self-contraction of the gel, which leads to the creation of a large number of shrinkage cracks and the expansion of pores (as shown in (B) of Figure 3(b-2)). This phenomenon is not obvious in Figure 3(a-2). The gel that formed by self-coagulation of liquid sodium silicate clearly further aggravated the shrinkage cracking characteristics because of the loss of free water and has insufficient strength, and cannot resist the formation and expansion of dry shrinkage cracks. Moreover, a large number of scattered particles (BA and GGBFS) can still be observed beneath the massive gel (as shown in (C) of Figure 3(b-2)). This further indicates that BA and GBFS particles are not involved in the self-coagulation reaction of liquid sodium silicate and they could not polymerize in an environment with low pH [34]. Since the strength of sample A-5 (only activated by liquid sodium silicate) only originates from the self-condensation of liquid sodium silicate, BA and GGBFS particles were only inert fillers. This also explains why the strengths of samples A-4 to A-6 (only activated by liquid sodium silicate) was clearly insufficient.

Figure 3(c-1) shows the microscopic reaction characteristics of sample A-8 after 3 h, which was excited both by NaOH and liquid sodium silicate (120 g). Although liquid sodium silicate was added, the surface of sample A-8 was not covered by liquid sodium silicate gels as was the case in sample A-5(b-1). Moreover, far less BA and GGBFS particles were scattered on the sample A-8 compared with sample A-3 (a-1). Almost all particles (BA and GGBFS) actively participated in the reaction and fused into the gel formed by liquid sodium silicate (as shown in (A) of Figure 3(c-1)). The excitation environment with high pH provided by NaOH fully stimulated the activities of BA and GGBFS, and the gels that formed through self-condensation of the liquid sodium silicate (as shown in (B) of Figure 3(c-1)) provide a carrier medium for the rapid polymerization of BA and GGBFS. Thus, the formation of polymerization products is accelerated and the polymerization rate is improved. Therefore, mixing both NaOH and liquid sodium silicate can supplement each other, further accelerated the condensation of sample A-8, and significantly improved the compressive strength [35]. Figure 3(c-2) shows the microscopic reaction characteristics of sample A-8 after 28 d, which was excited both by NaOH and liquid sodium silicate (120 g). A complete gel (as shown in (A) of Figure 3(c-2)) formed on the sample A-8, and the gel structure was compact without obvious cracks or pores, which was significantly better than the appearances of sample A-5 (b-2) and sample A-3 (a-2). Moreover, in sample A-8, scattering particles were almost not present, which indicates that these particles (BA and GGBFS) participate in the reaction to a very high degree and had formed many polymerization products [36]. Therefore, the microstructure of sample A-8, excited both by NaOH and liquid sodium silicate, was obviously better than that excited by one initiator alone.

Figure 3(d-1) shows the microscopic reaction characteristics of sample A-9 after 3 h, excited both by NaOH and liquid sodium silicate (140 g). With increasing liquid sodium silicate content, a large number of spicules and sheet crystals (as shown in (A) of Figure 3(d-1)), which were produced by the self-condensation of liquid sodium silicate, appeared on sample A-9. This phenomenon was not observed in sample A-8, see Figure 3(c-1). Moreover, sample A-9 (28 days, Figure 3(d-2)) was still covered with a small amount of crystals, formed by the self-coagulation reaction of liquid sodium silicate (as shown in (A) of Figure 3(d-2)). The amount of gels formed and their degree of compactness in sample A-9 was significantly weaker than in sample A-8, and the defects on sample A-9 were significantly more than on sample A-8 (28 days, Figure 3(c-2)). Because the liquid sodium silicate content was too high, as a result of the formation of too many crystals, the absorption ability of BA and GGBFS was exceeded, the excitation balance of both NaOH and liquid sodium silicate on BA and GGBFS was imbalanced, and the synergistic promotion between initiators was also obstructed [37]. Therefore, if too much liquid sodium silicate is added, the microstructure of sample A-9 will show many defects and the overall structure will not be compacted, which causes a decrease in strength.

#### 3.3.2. X-ray Diffraction Analysis

**Figure 4:**
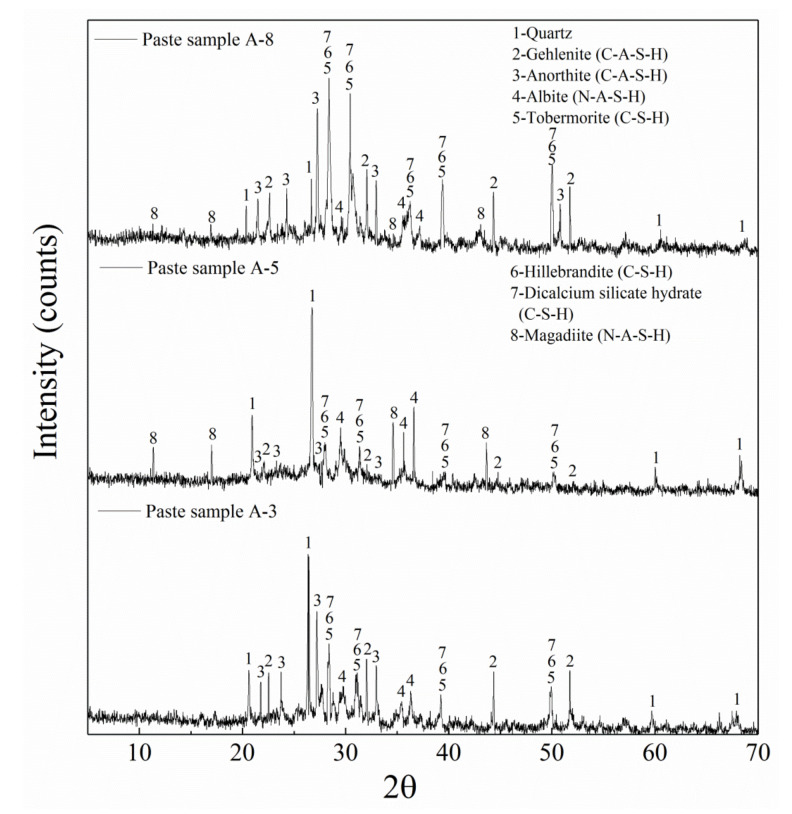
XRD (X-ray diffraction) analysis of paste samples A-3, A-5, and A-8.

Figure 4 shows the influence of different initiators on the formation and development of mineral crystals in paste samples A-3, A-5, and A-8 (all curing for 28 days).

Different kinds of mineral crystals formed in sample A-3, for which, NaOH was used as sole initiator at different dosages. The characteristic peaks of quartz (1; SiO_2_, and PDF #46-1045) appeared, which were marked as 1 and the intensity of the characteristic peak was very high. This indicates that sample A-3 contains a large number of quartz crystals. However, quartz crystals did not form by polymerization, and are mainly carried in BA and have low polymerization activity. At the same time, the characteristic peaks of gehlenite (2CaO∙Al_2_O_3_∙SiO_2_; PDF #35-0755) and anorthite (CaO∙Al_2_O_3_∙SiO_2_; PDF #41-1486) appeared in sample A-3, marked as 2 and 3, respectively. Gehlenite and anorthite are all calcium aluminum silicate minerals, which are considered to be calcium silicoaluminate hydrate (C-A-S-H) gels and are an important component for strength development. Moreover, characteristic peaks of tobermorite (Ca_5_(Si_6_O_16_)(OH_2_); PDF #45-1480), hillebrandite (Ca_2_(SiO_3_)(OH_2_); PDF #42-0538), and calcium silicate hydrate (Ca_3_SiO_5_∙3H_2_O; PDF #42-0551) also appeared in sample A-3, marked as 5, 6, and 7, respectively. Tobermorite, hillebrandite, and calcium silicate hydrate are all hydrated calcium silicate minerals, are considered to be C-S-H gels, and are also very important for the development of strength. In a strongly alkaline excitation environment, as supplied by NaOH, reactive calcium, silicon, and aluminum constantly melt out from BA and GGBFS and undergo polymerization reactions to produce a large number of critical polymerization products (such as C-S-H and C-A-S-H gels) [38]. This positively promoted an increase of compressive strength in sample A-3.

The mineral crystals that formed in sample A-5 (28 days) were significantly altered when only the initiator was changed from NaOH to liquid sodium silicate. The characteristic peak of quartz in sample A-5 showed no obvious change compared with sample A-3. However, sample A-5 did not show obvious characteristic peaks of gehlenite and anorthite (C-A-S-H gel), indicating that the formation of C-A-S-H gels in sample A-5 was severely affected compared with sample A-3. Moreover, the characteristic peaks of C-S-H gels (calcium silicate hydrate, hillebrandite, and tobermorite) also showed a sharp decrease compared with sample A-3. C-S-H and C-A-S-H gels play a key role in promoting the strength development, and the sharp decrease of these two characteristic peaks indicates that the degree of polymerization in sample A-5 was significantly reduced. The pH environment (pH = 11.55, see Table 2) that formed by liquid sodium silicate was too low (pH = 11.55, see Table 2) to reach a high alkali potential (pH 14). This high alkali potential in turn is needed to promote the precipitation of active substances from BA and GGBFS and could not promote the fusion of active substances to form C-S-H (which is favorable for the formation when the pH ranges between 13 and 14) and C-A-S-H gels (which is favorable for the formation when the pH exceeds 14) [39]. Therefore, the formation of C-S-H and C-A-S-H gels was significantly reduced in sample A-5 as a result of the significant deficiency of the precipitation of active substances, which seriously hindered the strength development [40]. Moreover, the obvious characteristic peaks of albite (Na_2_O·Al_2_O_3_·6SiO_2_; PDF #10-0393) and magadiite (Na_2_Si_14_O_29_·10H_2_O; PDF #42-1350) were found in sample A-5, marked as 4 and 8, respectively. Both mineral crystals were not found in sample A-3. Albite is a type of triclinic glassy crystal, which is also a sodium aluminosilicate and considered to be a kind of N-A-S-H gel. Liquid sodium silicate adds a large amount of active sodium and active silicon, but the reaction environment is in a state of severe shortage of both activated calcium and aluminum [41]. Such large amounts of active silicon can only bind to active sodium to form albite (N-A-S-H gel), which is why obvious characteristic peak of albite appeared in sample A-5. Moreover, part of the liquid sodium silicate rapidly self-condensed because of the loss of free water to form magadiite, which is also considered to be a kind of N-A-S-H gel. This is why the characteristic peak of magadiite was found in sample A-5. Therefore, the obviously shortened initial and final setting times of sample A-5 were caused by the formation of magadiite. The strength of the N-A-S-H gel is obviously inadequate and could not promote the further strength development of sample A-5 [42].

When both liquid sodium silicate and NaOH were used as initiators, the mineral crystals that formed in sample A-8 showed a marked improvement after 28 d of curing. The characteristic peaks of gehlenite and anorthite in sample A-8 achieved an obvious improvement compared with sample A-3 and A-5. Moreover, the characteristic peaks of tobermorite, hillebrandite, and calcium silicate hydrate in sample A-8 also showed a more significant improvement compared with sample A-3 and A-5, which indicates that the formation of C-A-S-H and C-S-H gels was also further improved. Under excitation of a strongly alkaline environment, supplied by NaOH (pH 14), the high alkali potential promotes the continuous precipitation of active substances (i.e., calcium, silicon, and aluminum) from BA and GGBFS and strongly promotes the reaction. Furthermore, the addition of liquid sodium silicate provides a sufficient amount of active silicon and increases the reaction rate. Therefore, under the coupling action of NaOH and liquid sodium silicate, the formed amount of C-A-S-H and C-S-H gel in specimen A-8 was significantly increased. The significant improvement of polymerization reaction efficiency, the rapid formation of gels, as well as the significant increase of gel production all further accelerated the coagulation of sample A-8 and improved its strength [43]. However, the characteristic peaks of the N-A-S-H gel (albite and magadiite) in sample A-8 exhibited a drastic decrease compared with samples A-3 and A-5. This suggested that the formation of albite and magadiite in sample A-8 was severely hindered. The rapid dissolution of active calcium and aluminum and the significant increase of dissolution promoted a fusion reaction of activated calcium and silicon, which seriously affected the polymerization of active silicon and sodium. Active calcium and sodium simultaneously compete for active silicon and active silicon preferably combines with calcium in a strongly alkaline environment (pH 14) [44]. More C-A-S-H and C-S-H gels are generated because of the existence of a large amount of active silicon, which actively combines with active calcium. This inevitably leads to a strong decrease of the combination rate of activated silica with sodium. Moreover, because of the increased active calcium content, the participating degree of liquid sodium silicate in the polymerization reaction is obviously increased. Inevitably, the amount of liquid sodium silicate that shows self-condensation decreased significantly. This is why the characteristic peaks of albite and magadiite decreased significantly.

### 3.4. Discussion

#### 3.4.1. Polymerization Mechanism Underlying NaOH Excitation

**Figure 5:**
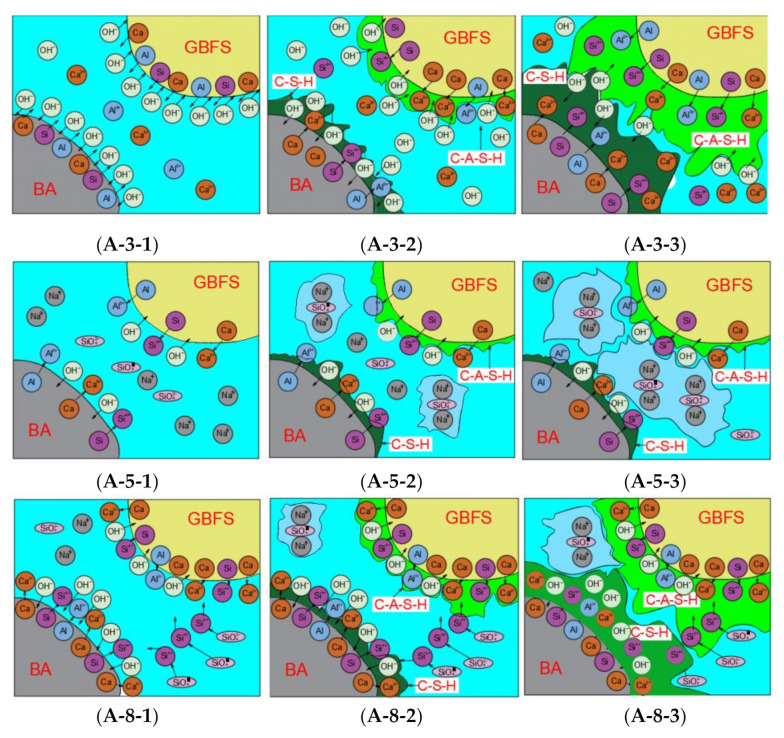
Polymerization mechanism of BA alkali-activated samples (**A-3**,**A-5**,**A-8**); (**A-3**) NaOH was used as sole initiator; (**A-5**) liquid sodium silicate was used as sole initiator; (**A-8**) both liquid sodium silicate and NaOH were used as initiator.

Figure 5 shows the setting and hardening mechanisms in response to different initiators in paste samples A-3, A-5, and A-8. The setting and hardening mechanism of sample A-3 is shown in Figure 5(A-3-1,A-3-2,A-3-3) where NaOH was used as the only initiator. After the sample preparation was completed, many OH^−^ molecules penetrated the interior of BA and GGBFS particles at high alkali potential and promoted active calcium, silicon, and aluminum to dissolute out of the BA and GGBFS particles in this strongly alkaline environment (as shown in Figure 5(A-3-1)). As the reaction continued, the polymerization product was generated continuously and the BA and GGBFS particles gradually bonded together by polymerization (as shown in Figure 5(A-3-2)). Moreover, with the increasing availability of OH^−^ molecules penetrating into the BA and GGBFS particles, the alkali potential inside the particles also increased gradually. This resulted in the simultaneous polymerization reaction of active substances inside the particle and the polymerization product is also generated inside the particle (as shown in Figure 5(A-3-2)) [45]. As the reaction continued, the gels that formed both inside and outside of the particles continued to increase, which leads to the complete convergence of the gels (both inside the outside of particles) into whole gels, which also integrated the particles. Furthermore, the continuous formation and development of the gels outside the particles constantly engulfed new particles and eventually formed a complete gel, which achieved the coagulation of the alkali-activated samples (as shown in Figure 5(A-3-3)). The coagulation of alkali-activated samples (which used NaOH as the only initiator) must first go through the slow infiltration of strong alkali solution and the dissolution of active substances [46]. Then, active substances polymerize to form gels. Finally, with the formation and further development of gels, the strength gradually built up and condensation of alkali-activated samples occurred. Therefore, samples that were only excited by NaOH tended to coagulate more slowly, but acceptable strength can be established because the polymerization reaction is thorough and produces high strength gels.

#### 3.4.2. Polymerization Mechanism Underlying Liquid Sodium Silicate Excitation

When the only initiator is converted from sodium hydroxide to liquid sodium silicate, the initial and final setting times and hardening mechanism of sample A-5 were completely different from sample A-3 (as shown in Figure 5(A-5-1,A-5-2,A-5-3)). During calcination (900 °C), the volatilization of combustible substances results in the mostly crisp and porous state of BA after calcination, which enables BA to easily absorb large amounts of free water. Moreover, liquid sodium silicate formed under high temperature and pressure and was supersaturated in water, which can therefore easily self-condensate when the supersaturated equilibrium is out of balance [47]. When BA and GGBFS particles contact liquid sodium silicate, small molecules of free water will quickly enter the BA pores; however, the larger molecules of sodium silicate cannot enter the BA, which aggravates the supersaturation state of liquid sodium silicate solution and thus disturbs the solution equilibrium (as shown in Figure 5(A-5-1)) [48]. Liquid sodium silicate was rapidly precipitated from the solution and crystallized to form magadiite and albite (N-A-S-H gel), which led to the rapid condensation of sample A-5 (this mechanism has been confirmed by SEM as shown in Figure 3(A-5-1) and by XRD analysis as shown in Figure 4). However, in sample A-5, BA, and GGBFS particles only act as inert fillings because, unlike sodium hydroxide, liquid sodium silicate cannot create a highly alkaline environment, and thus, cannot promote the precipitation and fusion of active substances (as shown in Figure 5(A-5-2)) [49]. Therefore, the self-condensation of liquid sodium silicate because of the loss of free water is the most important reason for the rapid coagulation of sample A-5. Since sample A-5 could not establish strength by formation of high strength gels (C-S-H and C-A-S-G-H), it could only establish and develop strength through the formation of low strength N-A-S-H gel (albite and magadiite) (as shown in Figure 5(A-5-3)). This led to the obviously insufficient sample A-5.

#### 3.4.3. Polymerization Mechanism Underlying Mixed Initiator Excitation

When a mix of both initiators was used, the excitations of NaOH and liquid sodium silicate were mutually promoting. Both NaOH and liquid sodium silicate can provide active sodium to the reaction environment, which leads to an excessively high content of active sodium in the reaction solution and disrupted the supersaturated equilibrium state of liquid sodium silicate [50]. This high Na^+^ concentration then accelerates the self-coagulation reaction of sodium silicate and promotes the formation of albite and magadiite (which has been confirmed by XRD analysis). Therefore, the initial and final setting times of sample A-8 were further shortened compared with sample A-5. Moreover, liquid sodium silicate also added considerable active silicon to the polymerization reaction environment (as shown in Figure 5(A-8-1)). The activated calcium immediately polymerized with active silicon to form C-S-H gels, which significantly promoted the production of high strength gels (as shown in Figure 5(A-8-2)) [51]. However, in sample A-3 (where NaOH was the sole initiator), reactive calcium, aluminum, and silicon had to go through a slower precipitation process before they could be released into the solution for fusion. This process clearly decreased the formation rate and amount of high strength of gels [52]. Moreover, the self-condensation reaction of liquid sodium silicate could pre-condense BA and GGBFS particles, this providing a good carrier for the formation of C-A-S-H and C-S-H gels. C-A-S-H and C-S-H gels are continuously formed both inside and outside of BA and GGBFS particles, which formed a complete and compact gel structure with sufficient polymerization reaction (as shown in Figure 5(A-8-3)) [53].

## 4. Conclusions

NaOH can provide a highly alkaline reaction environment, which is of the utmost importance for the polymerization and the formation as well as the development of high strength gels. The formation of C-A-S-H and C-S-H gels requires the processes of precipitation and refusion of active substances, which delays the condensation of alkali-activated samples.

Liquid sodium silicate alone cannot provide a sufficiently alkaline environment (pH 14), as it seriously affects the precipitation and refusion of activated calcium, silicon, and aluminum. Therefore, it can only compensate for the lack of strength by the self-condensation of liquid sodium silicate and the formation of a low-strength N-A-S-H gel. The self-condensation reaction of liquid sodium silicate (which forms magadiite) leads to the rapid condensation of alkali-activated samples.

When both NaOH and liquid sodium silicate were used as initiators, alkali-activated samples showed both rapid coagulation and high strength. The combination of NaOH and liquid sodium silicate will not affect their respective excitation effects but be mutually reinforcing.

## Figures and Tables

**Table 1:** Chemical composition of raw material (%).

	SiO_2_	CaO	Al_2_O_3_	Fe_2_O_3_	MgO	K_2_O	Na_2_O	Others	LOI
GGBFS	31.35	34.65	18.65	0.57	7.31	0.65	0.94	2.73	0.7
BA	53.82	14.44	14.18	6.18	3.26	2.52	2.24	0.61	1.62
Portland cement	26.55	59.34	7.77	1.62	2.68	1.5	0.31	1.25	3.2

GGBFS—Ground granulated blast furnace slag; BA—bottom-ash; LOI—Loss on ignition.

**Table 2:** Mix proportion of samples (kg/m^3^).

	I BA (60%)	II GGBFS (40%)	III Water	IV Sodium Silicate	Liquid-Solid Ratio	V NaOH	Sand	pH of Initiator
A-1	351.6	234.4	293	0	0.5	0	1758	10.02
A-2	351.6	234.4	293	0	0.5	15.63	1758	14.12
A-3	351.6	234.4	293	0	0.5	31.25	1758	14.43
A-4	351.6	234.4	208	130.2	0.5	0	1758	11.55
A-5	351.6	234.4	191	156.2	0.5	0	1758	11.55
A-6	351.6	234.4	174	182.3	0.5	0	1758	11.55
A-7	351.6	234.4	208	130.2	0.5	31.25	1758	14.49
A-8	351.6	234.4	191	156.2	0.5	31.25	1758	14.49
A-9	351.6	234.4	174	182.3	0.5	31.25	1758	14.49

Note: Solid = Ⅰ + Ⅱ, Liquid = Ⅲ + water in Ⅳ + Ⅴ. GGBFS—Ground granulated blast furnace slag; BA—bottom-ash.

**Table 3:** Setting time of BA alkali-activated paste samples (min/s).

Samples Number	Sodium Silicate	NaOH	Initial Setting Time	Final Setting Time
A-1	0	0	600 min	600 min
A-2	0	12	118 min	186 min
A-3	0	24	101 min	144 min
A-4	100	0	18 min	19 min 10 s
A-5	120	0	15 min	15 min 50 s
A-6	140	0	13 min	13 min 30 s
A-7	100	24	12 min	12 min 20 s
A-8	120	24	11 min	11 min 15 s
A-9	140	24	9 min	9 min 10 s

## Data Availability

Data is contained within the article.
